# Intraocular inflammatory cytokines in patients with neovascular age-related macular degeneration before and after initiation of intravitreal injection of anti-VEGF inhibitor

**DOI:** 10.1038/s41598-018-19594-6

**Published:** 2018-01-18

**Authors:** Tomohito Sato, Masaru Takeuchi, Yoko Karasawa, Toshio Enoki, Masataka Ito

**Affiliations:** 1 Department of Ophthalmology, National Defense Medical College, Tokorozawa, Saitama, Japan; 2 Department of Developmental Anatomy and Regenerative Biology, National Defense Medical College, Tokorozawa, Saitama, Japan; 3 Enoki Eye Clinic, Sayama, Saitama, Japan

## Abstract

Age-related macular degeneration (AMD) is a cause of blindness in people older than 50 years. Accumulating evidence indicates the involvement of systemic and local inflammation in the pathogenesis and progression of AMD. Aflibercept is an anti-vascular endothelial growth factor (VEGF) inhibitor, and intravitreal injection of aflibercept (IVA) is the approved treatments of neovascular AMD (nAMD), but the effect on inflammatory response remains unclear. The aim of our study was to investigate the profiles of inflammatory cytokines in the aqueous humor of nAMD patients before and after initiation of IVA. In nAMD patients, IP-10 level was significantly higher and IL-6 level was significantly lower compared with those of cataract patients as controls. Logistic regression analysis identified IP-10 as a positive factor and IL-6 a negative factor associated with the pathogenesis of nAMD. In addition, IP-10 level correlated positively with the mean thickness of macula in the central 1-mm diameter circle. After initiation of IVA, IP-10 level was further elevated, and correlated negatively with VEGF level. These data suggest that IP-10 plays a critical role as an antiangiogenic factor and at the same time an inflammatory factor in the pathogenesis and pathophysiology of nAMD eyes at onset and after IVA initiation.

## Introduction

Age-related macular degeneration (AMD) is a severe ocular disease affecting mainly elderly people, and is a major cause of blindness in people older than 50 years in developed countries^1^. Although the pathophysiology of AMD is not well understood, genetic variation, living environment and lifestyle have certain influence on the pathogenesis and progression of AMD^2^. AMD is broadly classified into two types according to the pathological mechanism; dry type and neovascular type^3^.

In neovascular AMD (nAMD), choroidal neovascularization (CNV) develops in the choroid, leading to sub- and intra-retinal macular edema, hemorrhage, and fibrosis responsible for visual decay^4^. Accumulating evidence indicates that systemic and ocular inflammation participates in the pathophysiology of nAMD^5,6^. Therefore, nAMD has been recognized not only as an exudative vascular event but also a chronic inflammatory disease in the retina and choroid^1,7^.

Vascular endothelial growth factor (VEGF) is a crucial mediator of angiogenesis and vascular permeability, and contributes to the development of choroidal neovascularization^5^. In mammals, the VEGF family consists of VEGF-A, VEGF-B, VEGF-C, VEGF-D and placenta growth factor (PlGF)^8^. VEGF-A and PlGF are signal proteins that promote angiogenesis and vascular leakage in the retina^9^. VEGF initiates biological effects by binding three transmembrane receptors; VEGF receptor (VEGFR)−1, VEGFR-2 and VEGFR-3^10^. VEGF receptors are expressed on various types of cells including vascular endothelial cells, pericytes, monocytes and macrophages^11^. VEGF-A recruits monocytes and macrophages by binding VEGFR-1 in the process of inflammatory neovascularization^12^. Since ocular fluid levels of VEGF increase markedly in nAMD patients, intravitreal injection of anti-VEGF agents has been the primary treatment for nAMD^4,13^. Aflibercept is an anti-VEGF agent and a fusion protein containing human VEGFR-1 and VEGFR-2 that bind VEGF-A, VEGF-B and PlGF. Intravitreal injection of aflibercept (IVA) is currently one of the promising treatments for nAMD^14^.

Aqueous humor and vitreous fluid reflect intraocular immune conditions of nAMD more directly than serum. Sampling of aqueous humor is easier and safer than sampling vitreous fluid. Furthermore, aqueous humor levels of cytokines have been proposed to reflect the levels in the vitreous cavity, and correlation between cytokines levels in aqueous humor and vitreous fluid has been reported^15,16^. Several papers that compared cytokine levels between nAMD patients and controls by nonparametric statistical tests indicated an association of aqueous humor cytokines with the pathogenesis of nAMD. Cha *et al*.^17^ reported that altered expression of insulin-like growth factor (IGF)-related molecules including IGF binding protein (IGFBP)-2 and IGF-1 may be involved in the pathogenesis of nAMD and suggested that these molecules are potential biomarkers for nAMD. Agawa *et al*.^5^ proposed that VEGF, angiogenin, interferon gamma-inducible protein (IP)-10, monocyte chemotactic protein (MCP)-1, macrophage inflammatory protein (MIP)-1β, and monokine induced by interferon γ (Mig) may be related to nAMD. Sakurada *et al*.^18^ also showed that IP-10 was associated with the pathogenesis of nAMD and polypoidal choroidal vasculopathy.

The purpose of this study was to investigate the association of aqueous humor cytokines with the pathogenesis of nAMD, and to evaluate the association of aqueous humor cytokine levels with clinical findings before and after initiation of IVA in eyes with nAMD.

## Methods

### Subjects

This prospective observational study enrolled 21 eyes of 21 nAMD patients (nAMD group) and 22 eyes of 17 cataract patients (control group). The study was performed at National Defense Medical College Hospital and Enoki Eye Clinic in Japan between August 1, 2013 and July 30, 2015. This study protocol was approved by the Ethics Committee of National Defense Medical College, and the procedures conformed to the tenets of the Declaration of Helsinki. Informed consent was obtained from all patients before enrolling in this study. The clinical characteristics of nAMD group and control group are summarized in Table 1. There were no significant differences in age and gender ratio between nAMD group and control group.

**Table 1:** Clinical characteristics of nAMD patients and controls.

Characteristic	nAMD patients (n = 21)	Control (n = 17)	*P* value
Age (year)	69.0 ± 10.2* (48 ∼ 8)	73.7 ± 9.43 (56 ∼ 90)	0.12
Gender (M/F)	15/6	6/11	0.06
phenotypes			
CNV Type I	4 (19^†^)		
CNV Type II	9 (43)		
PCV	8 (38)		
RAP	0 (0)		

nAMD: Neovascular age-related macular degeneration. CNV: Choroidal neovascularization. PCV: Polypoidal choroidal vasculopathy. RAP: Retinal angiomatous proliferation. *Mean ± standard deviation (range). ^†^Percentage.

Inclusion criteria for nAMD patients in this study were: (1) patients older than 50 years; (2) previously untreated CNV; (3) OCT showing intraretinal edema, subretinal fluid, or pigment epithelial detachment; and (4) absence of concurrent ocular diseases in the study eye, which had compromised or could have compromised vision and ocular condition. Exclusion criteria were: (1) clinical features suggesting that CNV was secondary to other causes such as pathologic myopia, trauma and hereditary diseases; (2) myopia greater than −6 diopter; (3) a history of treatment for nAMD, including intravitreal drug injection, photodynamic therapy, systemic or topical steroids; (4) previous intraocular surgery except cataract surgery, which had been performed more than 6 months before enrolment in the study.

### Diagnostics and Treatments

Diagnosis of nAMD was based on a full ophthalmological examination including best corrected visual acuity (BCVA) test using a decimal chart, slit-lamp biomicroscopy, dilated fundus examination, intraocular pressure (IOP) measurement, color fundus photography, fundus fluorescein angiography, fundus indocyanine green angiography and spectral-domain (SD) OCT (Cirrus HD-OCT; Carl Zeiss Meditec, Dublin, CA, USA). Neovascular AMD was classified according to the classification and diagnostic criteria of AMD^3,19^. Four eyes (19.0%) in the nAMD group was classified as type I neovascularization, 9 eyes (42.9%) as type II neovascularization, 8 eyes (38.0%) as polypoidal choroidal vasculopathy, and 0 eye (0%) as retinal angiomatous proliferation (Table 1). BCVA was converted to logMAR units for statistical analysis. For measurement of retinal thickness, a traditional Early Treatment Diabetic Retinopathy Study (ETDRS) grid containing three concentric rings 1, 3, and 6 mm in diameter was employed^20^. The mean retinal thicknesses of the macula in the central 1, 3 and 6 mm on the ETDRS grid were defined as CMT ≤ 1 mm, CMT ≤ 3 mm, and CMT ≤ 6 mm, respectively^21^. During the study period of 8 weeks, follow-up examinations were performed before the first IVA and after the third IVA.

### Intravitreal Injection of Aflibercept and Aqueous Humor Sample Collection

All nAMD patients were given intravitreal injection of 2 mg aflibercept every 4 weeks for 2 months (a total of three IVA) according to the protocol reported previously^5^. Before the first IVA (pre-IVA) and before the third IVA (post-IVA) in nAMD group, approximately 0.1 mL of undiluted aqueous humor was collected by performing an anterior chamber limbal paracentesis. Control samples of undiluted aqueous humor were obtained at the beginning of cataract surgery. The aqueous humor samples were transferred into sterile tubes and stored at −80 °C until processing. No complications associated with aqueous humor sampling occurred.

### Cytokine Measurements

Twenty-seven cytokines in aqueous humor samples were measured by a Bio-Plex multiplex assay (Bio-Plex Human Cytokine 27-plex panel; Bio-Rad, Hercules, CA, USA) and a multiplex bead analysis system (Bio-Plex Suspension Array System; Bio-Rad) according to manufacturers’ instructions with standards and samples in duplicate. The lower limits of detection for the 27 cytokines were as follows [mean (range)]: 4.18 (1.68–7.37) pg/ml for platelet derived growth factor BB (PDGF-BB); 1.25 (0.53–1.99) pg/ml for IL-1β; 6.03 (5.73–6.47) pg/ml for IL-1 receptor antagonist (IL-1ra); 1.07 (0.93–1.21) pg/ml for IL-2; 0.48 (0.21–1.15) pg/ml for IL-4; 1.41 (1.15–1.62) pg/ml for IL-5; 1.83 (1.38–2.28) pg/ml for IL-6; 1.62 (0.98–2.18) pg/ml for IL-7; 2.25 (1.91–2.58) pg/ml for IL-8; 1.60 (0.57–2.32) pg/ml for IL-9; 2.14 (2.14–2.15) pg/ml for IL-10; 2.37 (2.27–2.44) pg/ml for IL-12; 1.25 (0.45–2.03) pg/ml for IL-13; 1.43 (1.37–1.49) pg/ml for IL-15; 3.56 (1.62–5.93) pg/ml for IL-17A; 3.05 (1.26–6.8) pg/ml for eotaxin; 2.70 (0.78–3.85) pg/ml for basic fibroblast growth factor (bFGF); 2.16 (1.90–2.41) pg/ml for granulocyte colony-stimulating factor (G-CSF); 2.94 pg/ml (0.63–4.01 pg/ml) for Granulocyte-macrophage colony-stimulating factor (GM-CSF); 9.80 (5.06–21.21) pg/ml for IFN-γ; 8.34 (7.36–9.18) pg/ml for IP-10; 1.49 (1.29–1.67) pg/ml for MCP-1; 0.83 (0.28–1.05) pg/ml for MIP-1α; 2.18 (0.53–3.77) pg/ml for MIP-1β; 2.09 (1.03–4.11) pg/ml for regulated on activation, normal T-cell expressed and secreted (RANTES); 4.76 (4.05–5.64) pg/ml for tumor necrosis factor α (TNFα); and 2.04 (1.63–2.83) pg/ml for VEGF. Levels of aqueous humor cytokines below detectable levels were treated as 0 for statistical analysis^5^.

### Statistical Analysis

Statistical analyses were performed using the statistic add-in software for Excel (SSRI Co., Ltd., Tokyo, Japan). Data are expressed as mean ± standard deviation (SD). Yates’ chi-squared test (for n < 10) or Fisher’s exact test (for n < 4) was used to compare categorical variables. Mann-Whitney *U* test was used to compare aqueous humor levels of cytokines between nAMD group and control group. Wilcoxon signed rank test was used to compare aqueous humor levels of cytokines before the first IVA (pre-IVA) and before the third IVA (post-IVA) in nAMD group. Spearman’s correlation analysis was used to evaluate the relationship between numerical data. To examine the association of elevated or decreased cytokines with the pathogenesis of nAMD, logistic regression analysis was performed using nAMD or not as response variable and cytokines with high detectable rates of over 80% in pre-IVA of nAMD and control groups and significant differences between them as explanatory valuables. A *P* level less than 0.05 was considered to be statistically significant.

### Data Availability

All data presented in the present study are available from the corresponding author on reasonable request.

## Results

**Figure 1:**
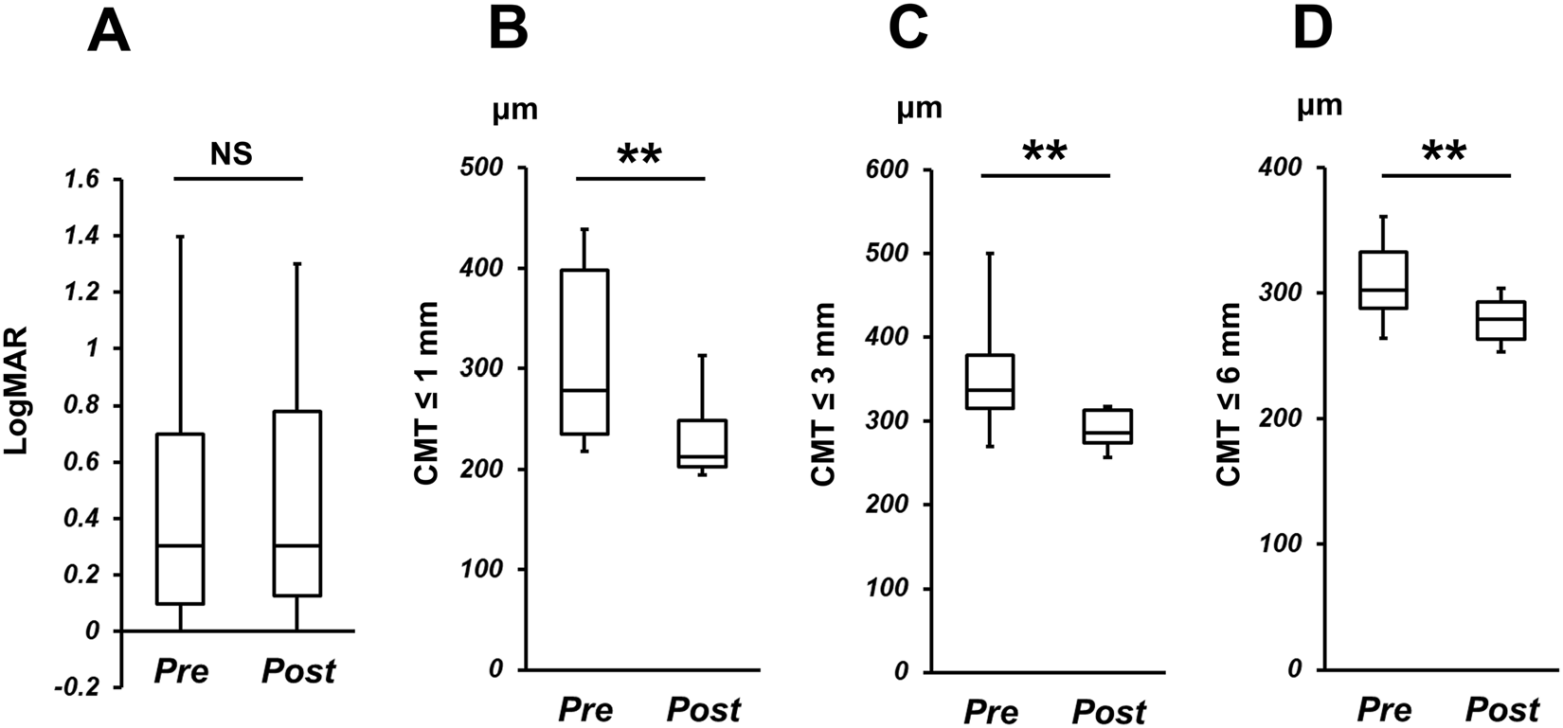
LogMAR and central macular thickness (CMT). (**A**) LogMAR, (**B**) CMT ≤ 1 mm, (**C**) CMT ≤ 3 mm, and (**D**) CMT ≤ 6 mm in control group (Cont), and in nAMD group before (nAMD pre) and after initiation of IVA therapy (nAMD post). NS: Not significant, **P < 0.01.

The profiles of logMAR and mean macular thickness in nAMD group before and after initiation of IVA therapy are shown in Fig. 1. LogMAR was 0.51 ± 0.60 before IVA and 0.48 ± 0.67 after IVA, and there was no significant difference between them (*P* = 0.33). On the other hand, CMT ≤ 1 mm, CMT ≤ 3 mm, and CMT ≤ 6 mm were 345 ± 201 µm, 384 ± 150 µm, and 321 ± 63.7 µm before IVA, and were 256 ± 129 µm, 307 ± 82.3 µm, and 284 ± 33.0 µm after IVA, respectively. There were significant differences in CMT ≤ 1 mm, CMT ≤ 3 mm, and CMT ≤ 6 mm before and after IVA (*P* = 2.11 × 10^−4^, 2.98 × 10^−5^, and 3.48 × 10^−5^, respectively).

### Aqueous Humor Cytokine Levels in Control Group and in nAMD Group Before and After IVA Therapy

The profiles of aqueous humor levels of cytokines detected in nAMD group before and after the initiation of IVA therapy, and in control group are shown in Table 2. In nAMD group before IVA, IL-6 level was significantly lower (*P* = 0.0029), but Eotaxin, IP-10 and VEGF levels were significantly higher (*P* = 0.035, *P* < 0.001 and *P* = 0.029, respectively) compared to control group. Even after initiation of IVA, IL-6 level remained significantly lower (*P* = 0.011), and Eotaxin and IP-10 levels were significantly higher (*P* = 0.043 and *P* < 0.001, respectively) compared to control group. When comparing pre-IVA and post-IVA levels in nAMD group, IL-6 and IP-10 levels were elevated (*P* = 0.0098 and *P* = 0.0026, respectively), while IL-12 and VEGF levels were reduced remarkably (both *P* < 0.001). In logistic regression analysis using nAMD or not as response variable and aqueous humor levels of IL-6, IP-10 and VEGF in control and pre-IVA of nAMD groups as explanatory valuables, it was also demonstrated that pre-IVA IP-10 and VEGF levels were significant higher (*P* = 0.018 and *P* = 0.043, respectively) and IL-6 level was significant lower (*P* = 0.043) in nAMD group than those in control group (Table 3).

**Table 2:** Aqueous humor levels of cytokines in controls and in nAMD patients before or after initiation of IVA therapy.

	nAMD (n = 21)				Controls (n = 17)			*P* value	
	pre-IVA		post-IVA						
	Detectable	Level	Detectable	Level	Detectable	Level	Pre IVA	Post IVA	Pre IVA
	samples*	mean ± SD	samples	mean ± SD	samples	mean ± SD	vs Control	vs Control	vs Post IVA
IL-1rα	0 (0)*	0	1 (4.76)	0.70 ± 3.23^†^	1 (5.88)	0.77 ± 3.19	0.381	0.483	0.164
IL-6	16 (76.2)	6.51 ± 5.24	18 (85.7)	12.6 ± 13.7	15 (88.2)	78.2 ± 100	0.0029	0.011	0.0098
IL-7	21 (100)	10.9 ± 3.95	21 (100)	12.4 ± 6.67	17 (100)	13.5 ± 12.8	0.465	0.397	0.219
IL-8	12 (57.1)	6.00 ± 6.69	14 (66.7)	10.3 ± 11.7	11 (64.7)	6.43 ± 6.84	0.448	0.177	0.087
IL-9	1 (4.76)	0.087 ± 0.40	1 (4.76)	0.18 ± 0.81	1 (5.88)	0.10 ± 0.41	0.483	0.483	0.328
IL-12	20 (95.2)	12.1 ± 5.79	0 (0)	0	12 (70.6)	10.4 ± 9.11	0.268	3.44 × 10^−4^	4.45 × 10^−5^
IL-13	12 (57.1)	1.97 ± 2.22	11 (52.4)	1.92 ± 2.59	11 (64.7)	2.25 ± 2.65	0.448	0.316	0.398
Eotaxin	11 (52.4)	3.76 ± 4.17	11 (52.4)	3.66 ± 4.04	1 (5.88)	1.22 ± 2.39	0.035	0.043	1
βFGF	0 (0)	0	1 (4.76)	0.76 ± 3.49	1 (4.55)	0.71 ± 2.92	0.381	0.483	0.164
G-CSF	1 (4.76)	0.28 ± 1.27	0 (0)	0	0 (0)	0	0.402	—	0.164
IP-10	21 (100)	755 ± 645	21 (100)	1844 ± 2719	17 (100)	273 ± 260	3.00 × 10^−4^	6.85 × 10^−5^	0.0026
MCP-1	21 (100)	229 ± 155	21 (100)	182 ± 143	17 (100)	204 ± 112	0.381	0.184	0.085
MIP-1α	4 (19.0)	0.41 ± 0.91	5 (23.8)	0.64 ± 1.29	4 (23.5)	0.96 ± 2.35	0.4	0.494	0.122
MIP-1β	21(100)	37.2 ± 15.6	21 (100)	38.3 ± 19.6	17 (100)	37.2 ± 31.1	0.112	0.197	0.372
VEGF	21 (100)	228 ± 176	2 (9.52)	1.08 ± 3.46	17 (100)	132 ± 54.2	0.029	3.14 × 10^–6^	2.98 × 10^–5^

nAMD: Neovascular age-related macular degeneration. IVA: Intravitreal injection of aflibercept. ^*^Number (%) of samples with detectable cytokine. ^†^Mean ± standard deviation (pg/ml).

**Table 3:** Logistic regression analysis of the association of nAMD with cytokines in aqueous humor.

	OR	95% CI	*P* value
IL-6	0.920	(0.848–0.997)	0.043
IP-10	1.004	(1.0007–1.007)	0.018
VEGF	1.009	(1.0003–1.019)	0.043

nAMD: Neovascular age-related macular degeneration. OR = Odds ratio. CI = Confidence interval.

### Correlation of logMAR and Central Macular Thickness with Aqueous Humor IL-6, IP-10 and VEGF Levels in nAMD Patients

**Figure 2:**
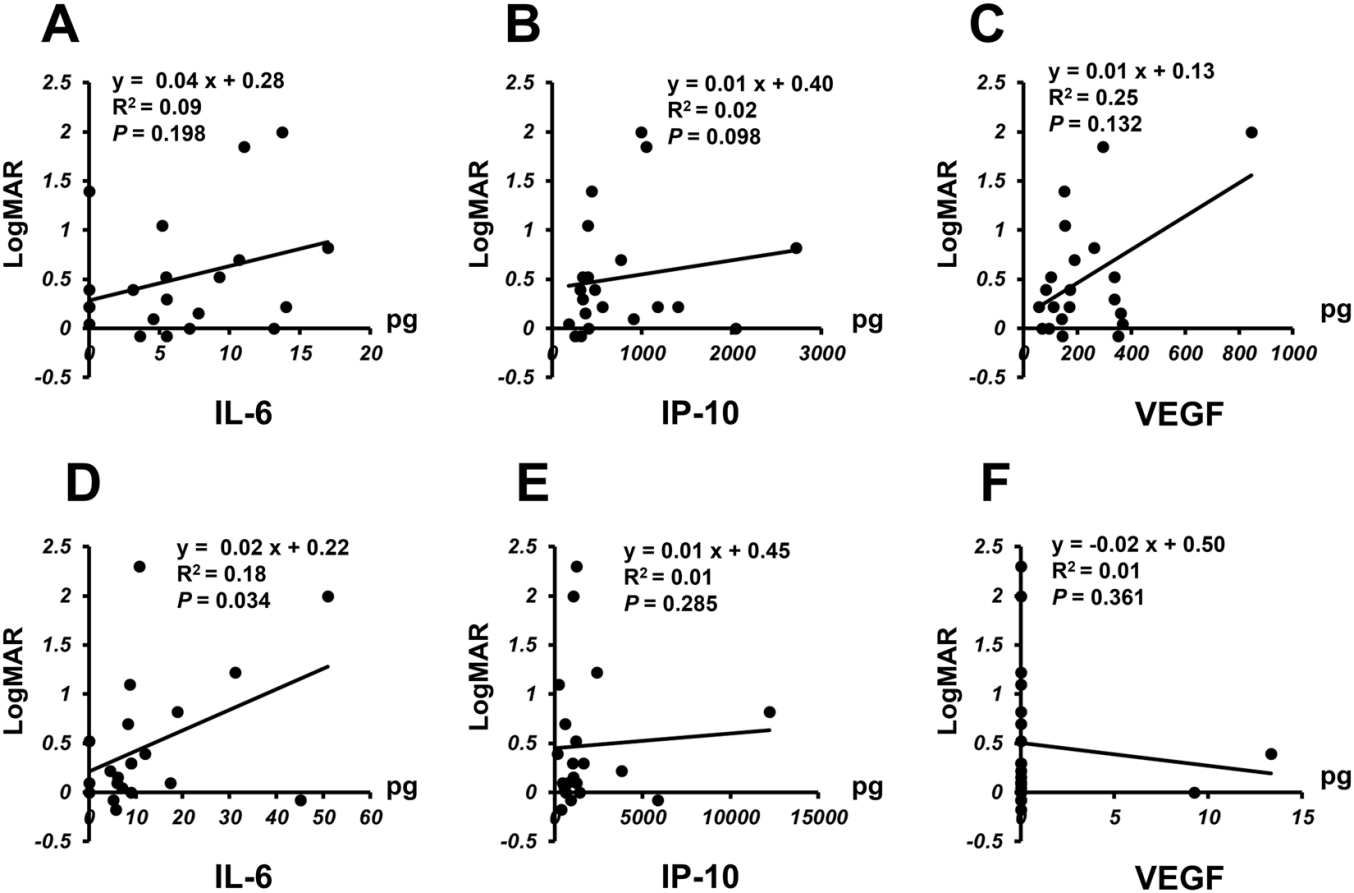
Relationship between logMAR and aqueous humor level of IL-6, IP-10 or VEGF in nAMD group before and after initiation of IVA therapy. Correlation between logMAR and aqueous humor level of IL-6, IP-10 or VEGF in nAMD group before (pre-IVA) (**A**–**C**) and after initiation of IVA therapy (post-nAMD) (**D**–**F**).

Based on the results of Table 2, we next examined the associations between clinical findings and aqueous humor IL-6, IP-10, and VEGF levels before and after initiation of IVA therapy in nAMD group. Figure 2 shows correlation between logMAR and aqueous humor level of IL-6, IP-10, or VEGF before and after initiation of IVA. Before IVA therapy, there was no significant correlation between logMAR and IL-6, IP-10, or VEGF level (Fig. 2A–C). However, a positive correlation (*P* = 0.034) between IL-6 level and logMAR was observed after initiation of IVA therapy (Fig. 2D), although there was no significant correlation between logMAR and IP-10 or VEGF (Fig. 2E and F).

**Figure 3:**
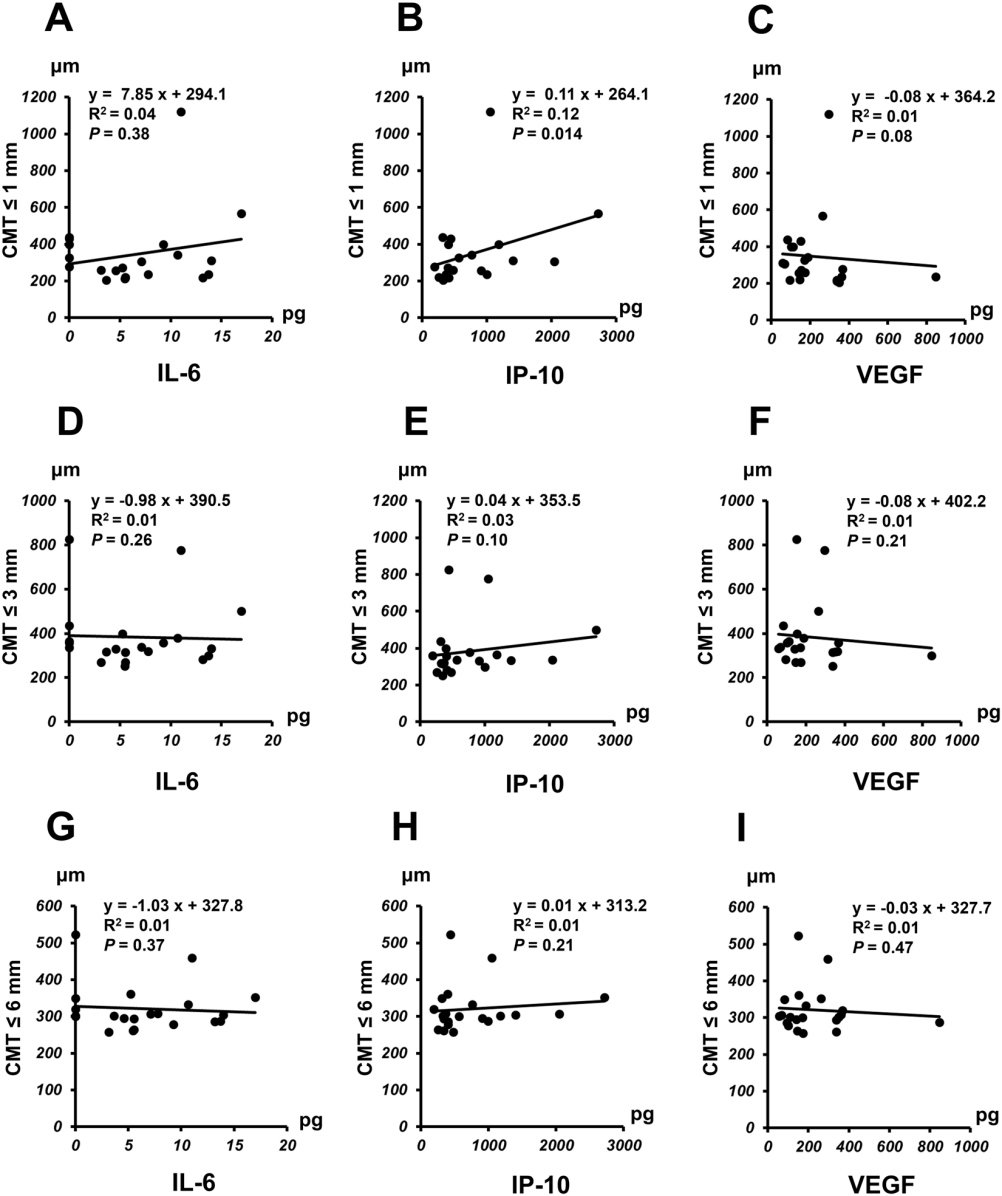
Relationship between central macular thickness (CMT) and aqueous humor level of IL-6, IP-10 or VEGF in nAMD group before initiation of IVA therapy. Correlation of CMT ≤ 1 mm (**A**–**C**), CMT ≤ 3 mm (**D**–**F**), or CMT ≤ 6 (**G**–**I**) mm with pre-IVA aqueous humor level of IL-6, IP-10 or VEGF in nAMD group.

**Figure 4:**
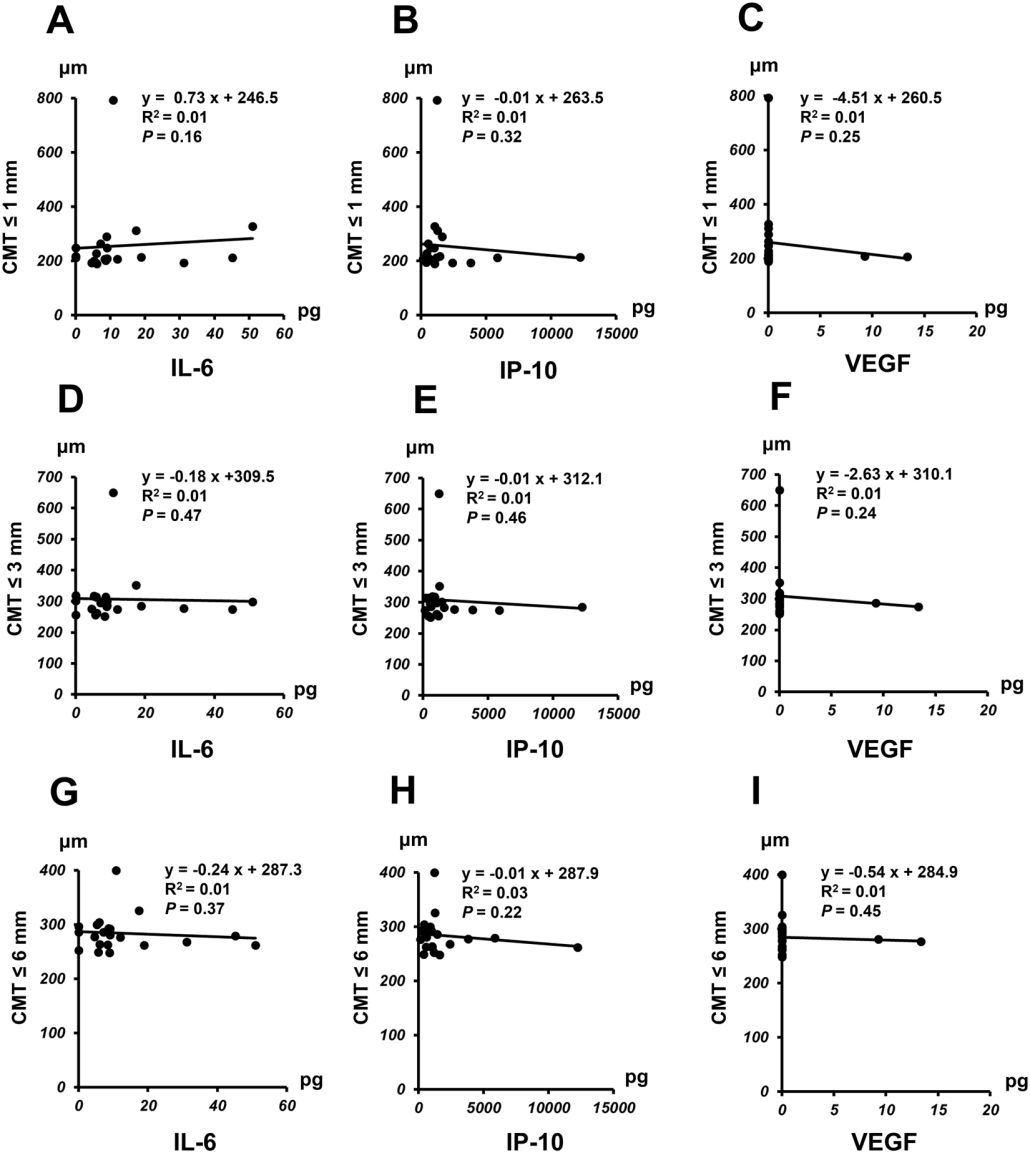
Relationship between central macular thickness (CMT) and aqueous humor level of IL-6, IP-10 or VEGF in nAMD group after initiation of IVA therapy. Correlation of CMT ≤ 1 mm(**A**–**C**), CMT ≤ 3 mm (**D**–**F**), or CMT ≤ 6 mm (**G**–**I**) with post-IVA aqueous humor level of IL-6, IP-10 or VEGF in nAMD group.

Correlation of CMT with aqueous humor levels of IL-6, IP-10, or VEGF in nAMD group before IVA therapy is shown in Fig. 3 and the correlation after IVA therapy is presented in Fig. 4. A positive correlation was observed between pre-IVA level of IP-10 and CMT ≤ 1 mm (*P* = 0.014) (Fig. 3B), although no significant correlation was found between pre-IVA IL-6 or VEGF level and CMT ≤ 1 mm (Fig. 3A and C). There was no significant correlation between pre-IVA level of IL-6, IP-10 or VEGF and CMT ≤ 3 mm or CMT ≤ 6 mm (Fig. 3D–I). For the analysis of post-IVA levels, there was no significant correlation between IL-6, IP-10, or VEGF level and CMT < 1 mm, CMT ≤ 3 mm, or CMT ≤ 6 mm (Fig. 4A–I).

### Correlation among Levels of IL-6, IP-10 and VEGF in nAMD Patients at Pre-IVA and Post-IVA

**Figure 5:**
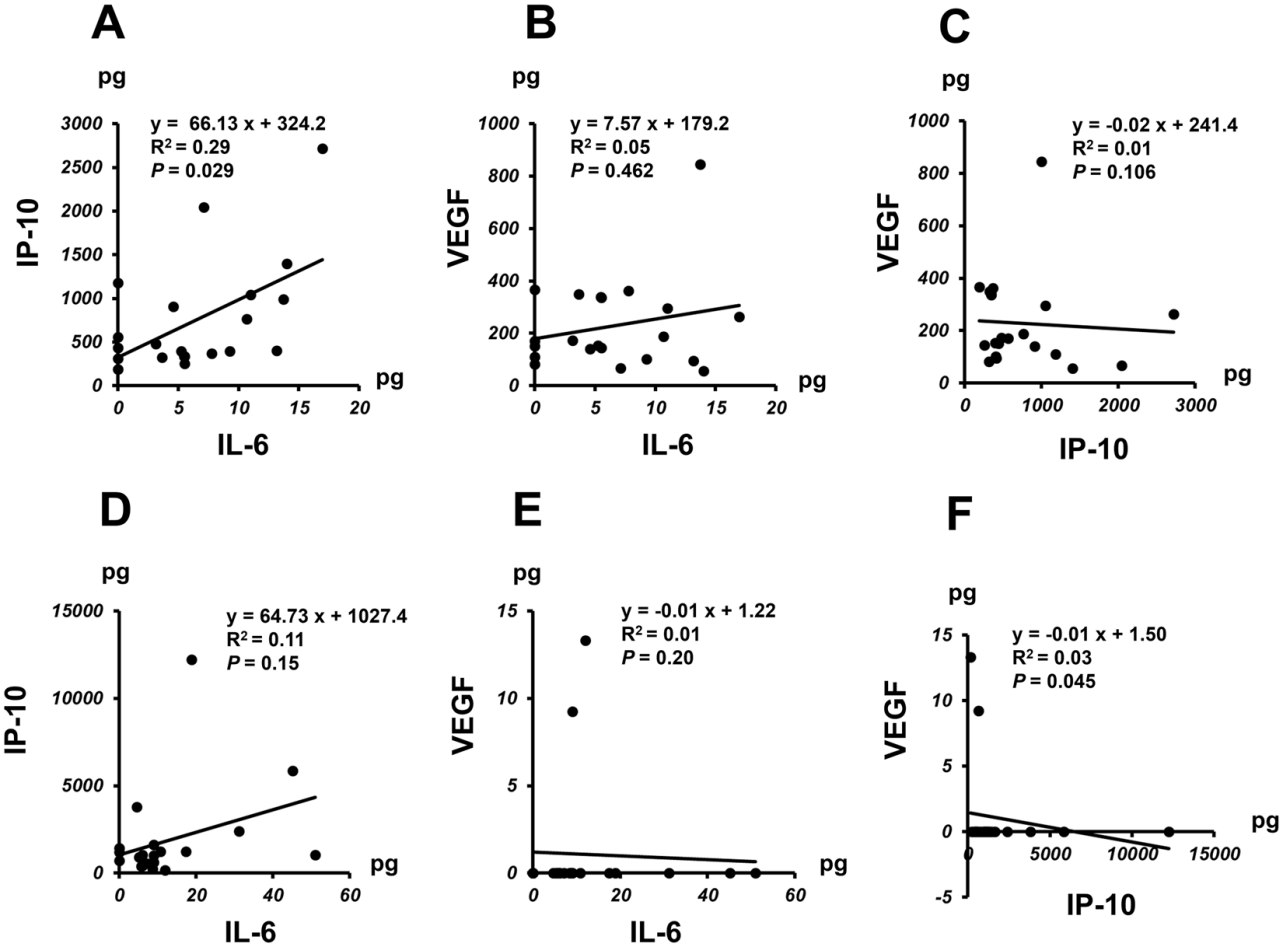
Association among levels of IL-6, IP-10 and VEGF in nAMD group before and after initiation of IVA therapy. Correlation among pre-IVA aqueous humor levels of IL-6, IP-10 and VEGF and at post-IVA levels (**D**–**F**) in nAMD group.

Figure 5 shows the correlation among pre-IVA and post-IVA levels of IL-6, IP-10, and VEGF in nAMD group. For the comparisons of pre-IVA levels, a positive correlation was observed between IL-6 level and IP-10 level (*P* = 0.029) (Fig. 5A), but no significant correlation was detected between VEGF level and IL-6 or IP-10 level (Fig. 5B and C). In post-IVA, there was a negative correlation between IP-10 level and VEGF level (*P* = 0.045), but no significant correlation between IL-6 level and IP-10 or VEGF level (Fig. 5D–F).

## Discussion

The major findings of the present study were as follows. (1) High levels of VEGF and IP-10, and low level of IL-6 were the characteristic profile of inflammatory cytokines in aqueous humor of nAMD patients, (2) IVA reduced VEGF level and increased IL-6 level, but further increased IP-10 level in aqueous humor, with a negative correlation between IP-10 and VEGF levels. (3) Increased IL-6 level in aqueous humor of nAMD patients after initiation of IVA treatment negatively correlated with BCVA, and IP-10 level correlated with CMT ≤ 1 mm before but not after initiation of IVA.

AMD is a leading cause of irreversible blindness in people aged 50 years or older living in the developed world^1^. Genetic variation, living environment and lifestyle have been known to be risk factors in the pathogenesis and progression of AMD^1^. Previous studies on nAMD patients showed that complement components in plasma were activated, and the levels of proinflammatory cytokines in aqueous humor were significantly higher compared with those in cataract patients as controls^5,6,22,23^. Therefore, nAMD has been recognized not only an exudative vascular disease but also a chronic inflammatory disease in the retina and choroid^1,7,24^.

The aqueous humor reflects the immunological conditions of the eye more directly than serum, and has been used as the major intraocular sample because it can be collected repeatedly and more conveniently than vitreous fluid. Boyd *et al*.^25^ reported that aqueous humor VEGF level correlated significantly with that of vitreous fluid. Therefore, we selected aqueous humor cytokines as biomarkers that indicate the immunological features of eyes with nAMD.

In this study, BCVA did not improve significantly, but tended to recover by 2 sessions of IVA in nAMD patients, although CMT was reduced significantly. VIEW 2 study reported that IVA every 4 weeks improved visual acuity by approximately 7 ETDRS letters after 8 weeks, and the degree of BCVA improvement was almost equivalent to a decrease of 0.14 logMAR^26^. The numbers of subjects enormously affect statistical analysis, and VIEW 2 study is a much larger clinical study compared with the present study. In addition, the inclusion criteria of VIEW 2 study are different from our study. In VIEW 2 study, the inclusion criteria are as follows: (1) CNV comprised at least 50% of total lesion size; (2) BCVA before IVA was within a range of 25 to 73 ETDRS letters, almost equivalent to logMAR 0.301 to 0.164. In our study, the CNV lesion size and BCVA were not restricted, and nAMD patients with a wider range of conditions are enrolled in this study as shown in Fig. 2. Therefore, two sessions of IVA might be not sufficient in treatment duration and/or treatment frequency to improve BCVA significantly.

Our study showed that IP-10 was elevated in the aqueous humor of nAMD patients, and IVA further increased the IP-10 level. IP-10 is secreted by leukocytes such as monocytes and macrophages, and by non-leukocytes including endothelial cells and smooth muscle cells^27^. Regarding response to neovascularization, IP-10 has antiangiogenic and antifibrotic actions^28,29^. Bodnar *et al*.^30^ reported that IP-10 inhibited VEGF-induced endothelial cell motility and tube formation *in vitro*. In postmortem eyes with early AMD, IP-10 was strongly expressed in neovascular endothelial cells and connective tissue matrix associated with CNV^31^. Boulday *et al*.^32^ reported that VEGF induced over-expression of IP-10 in endothelial cells *in vitro* and *in vivo*. Findings of the present study and previous reports thus suggest that aflibercept may induce IP-10 production that exhibits antiangiogenic effects to inhibit the development of CNV at the onset of nAMD.

Higher level of IP-10 in aqueous humor of nAMD was associated with greater CMT before IVA therapy, although decrease of CMT following IVA abolished the association. Sakamoto *et al*. also measured IP-10 level in the aqueous humor of nAMD patients before first and third intravitreal injection of ranimizumab (IVR), and found that the aqueous humor level of IP-10 in nAMD patients was higher than that of controls although IP-10 was not increased after the treatment with IVR^33^. In addition, they indicated that IP-10 level in the aqueous humor was associated with initial CRT by multiple regression analysis, as well as our present results.

IP-10 is a specific chemotactic factor for Th1 cells, and activates Th1 cell-mediated immune response via binding the common CXC chemokine receptor (CXCR)3^27,34^. Previous *in vitro* and *in vivo* studies reported that CCR3 and CXCR3 were involved in the development of nAMD, and CCR2 and CX3CR1 were associated with drusen formation in early AMD^35–38^. Examination of postmortem eyes with nAMD revealed overexpression of helper T-cell-related cytokines in the retina^31,39^. Singh *et al*.^40^ suggested the involvement of the adaptive immune system in AMD, and proposed AMD as a systemic disease. Therefore, it is possible that low-grade inflammation related to high level of IP-10 induces increase in central macular thickness in nAMD, independent of the development of CNV.

On the other hand, angiogenesis is arrested during the regenerative phase of wound healing^30^. After IVA treatment, CNV may also be attenuated, and the regenerative and remodeling process may be initiated in the CNV-damaged retina. IP-10 is produced by vascular endothelial cells late in the regenerative phase and CXCR3 has also been found to be expressed on human endothelial cells^30,41,42^. CXCR3 ligands including IP-10 inhibit chemotaxis of fibroblasts^43^. Therefore, we speculate that the IP-10 overexpressed in the damaged retina in IVA-treated eyes may suppress the progression of fibrosis in the retina during the regenerative and remodeling phase.

IL-6 functions both as a pro-inflammatory and an anti-inflammatory cytokine^44^. IL-6 directly or indirectly induces numerous angiogenic and proinflammatory cytokines including VEGF^45^. AMD is a chronic inflammatory disease and is under a para-inflammatory state in which macrophages and T cells work to maintain homeostasis in the retina and choroid^46,47^. Agawa *et al*.^5^ showed that aqueous humor IL-6 level in nAMD patients tended to be lower compared with that in cataract patients. Down-expression of IL-6 is conceivably a part of homeostatic response in nAMD patients to suppress CNV development and vascular permeability induced by VEGF, although some contradictory results have been reported^48,49^. Furthermore, aqueous humor IP-10 level correlated positively with IL-6 level in nAMD eyes, suggesting that IP-10 and IL-6 act mutually to counteract the excessive angiogenetic and anti-angiogenetic responses in the eyes of nAMD.

IVA treatment reduced VEGF and IL-12 levels remarkably. VEGF-A is known to recruit macrophages and monocytes, and VEGF-R1 is expressed abundantly on the membrane of these cells^50,51^. IL-12 is expressed by antigen-presenting cells such as dendritic cells, monocytes, macrophages and B cells^52^. Although VEGF level in the aqueous humor might be measured lower by contamination of aflibercept after IVA^53^, it is possible that blocking ligation of VEGF-A and VEGF-R1 by IVA results in inactivation of macrophages and monocytes including IL-12 production by these cells.

Our study demonstrated that IL-6 level was elevated by IVA treatment and correlated negatively with BCVA. During IVA treatment, VEGF level was clearly suppressed and the level was extremely low compared with that of controls. IL-6 simultaneously induces VEGF production and causes inflammation^45^. Calvo *et al*.^54^ reported that ranibizumab treatment with dexamethasone intravitreal implant as adjunct therapy reduced persistent intra/sub-retinal fluid on OCT and maintained BCVA in ranibizumab-resistant patients with longstanding nAMD. Therefore, we hypothesize that elevation of IL-6 level was induced in response to marked decrease in VEGF by IVA, and collateral inflammation may deteriorate BCVA, although BCVA depends on several optical and neural factors, and the relation between BCVA and IL-6 is complicated.

The present study has several limitations. First, the number of nAMD patients was too small to perform subgroup analysis for various phenotypes of nAMD. In the future, a large-scale multi-institutional survey may reveal various immunological characteristics for different phenotypes of nAMD. Second, the observation period of our study was short, and the evaluation of kinetics of aqueous humor cytokines was limited to the initial phase of IVA therapy. Longer follow-up is needed to allow better understanding of the pathophysiology in the maintenance phase of IVA.

The strength of this study was that during the observation period, no enrolled patient dropped out of the study or underwent cataract surgery that would change BCVA and influence retinal thickness and cytokine levels. Therefore, aqueous humor samples were collected under relatively unbiased conditions and thus allowed precise evaluation of the association of proinflammatory cytokines and pathophysiology of nAMD before and after the initiation of IVA.

In conclusion, the present results demonstrate that in addition to VEGF, IP-10 and IL-6 play crucial roles in the pathophysiology and pathogenesis of nAMD, and that IVA decreases intraocular VEGF level but increases IP-10 and IL-6 levels.
